# Birth weight and growth from infancy to late adolescence in relation to fat and lean mass in early old age: findings from the MRC National Survey of Health and Development

**DOI:** 10.1038/ijo.2013.115

**Published:** 2013-07-16

**Authors:** D Bann, A Wills, R Cooper, R Hardy, A Aihie Sayer, J Adams, D Kuh

**Affiliations:** 1 MRC Unit for Lifelong Health and Ageing, Division of Population Health, University College London, London, UK; 2 MRC CAiTE, School of Social and Community Medicine, University of Bristol, Bristol, UK; 3 Academic Geriatric Medicine, MRC Lifecourse Epidemiology Unit, University of Southampton, Southampton, UK; 4 Clinical Radiology and Manchester Academic Health Science Centre (MAHSC), Manchester Royal Infirmary, University of Manchester, Manchester, UK

**Keywords:** abdominal obesity, growth, birth weight, body composition, muscle mass

## Abstract

**Objective:**

High birth weight and greater weight gain in infancy have been associated with increased risk of obesity as assessed using body mass index, but few studies have examined associations with direct measures of fat and lean mass. This study examined associations of birth weight and weight and height gain in infancy, childhood and adolescence with fat and lean mass in early old age.

**Subjects:**

A total of 746 men and 812 women in England, Scotland and Wales from the MRC National Survey of Health and Development whose heights and weights had been prospectively ascertained across childhood and adolescence and who had dual energy X-ray absorptiometry measures at age 60–64 years.

**Methods:**

Associations of birth weight and standardised weight and height (0–2 (weight only), 2–4, 4–7, 7–11, 11–15, 15–20 years) gain velocities with outcome measures were examined.

**Results:**

Higher birth weight was associated with higher lean mass and lower android/gynoid ratio at age 60–64 years. For example, the mean difference in lean mass per 1 standard deviation increase in birth weight was 1.54 kg in males (95% confidence interval=1.04, 2.03) and 0.78 kg in females (0.41, 1.14). Greater weight gain in infancy was associated with higher lean mass, whereas greater gains in weight in later childhood and adolescence were associated with higher fat and lean mass, and fat/lean and android/gynoid ratios. Across growth intervals greater height gain was associated with higher lean but not fat mass, and with lower fat/lean and android/gynoid ratios.

**Conclusion:**

Findings suggest that growth in early life may have lasting effects on fat and lean mass. Greater weight gain before birth and in infancy may be beneficial by leading to higher lean mass, whereas greater weight gain in later childhood and adolescence may be detrimental by leading to higher fat/lean and android/gynoid ratios.

## Introduction

Fat and lean mass influence health and physical functioning. High fat mass is associated with a range of adverse health outcomes, including type 2 diabetes, hypertension, stroke, impaired physical functioning and higher rates of mortality.^1^ Low lean mass, an indicator of low skeletal muscle mass, is associated with lower physical performance levels,^2, 3^ adverse glucose metabolism^4^ and low bone mineral content.^5^ Low muscle mass is also an essential component of the most widely used definitions of sarcopenia,^6, 7^ a condition of increasing public health concern.^6^

In addition to evidence that contemporaneous factors such as physical activity and diet influence adult fat and lean mass,^8, 9^ there is growing evidence that factors such as growth during gestation, infancy, childhood and adolescence may also have a role. High birth weight^10^ and rapid weight gain in infancy^11, 12^ have been consistently shown to be associated with increased risk of obesity in adulthood, measured by body mass index (BMI). However, studies using BMI are unable to elucidate whether associations reflect influences of growth on fat mass, lean mass or both. For example, although an association between high birth weight and high BMI suggests an association with fat mass, some studies have found that birth weight is positively associated with lean mass, with either weak or no association found between birth weight and fat mass.^13, 14, 15^ Relatively few studies have examined associations between early growth and direct measures of fat and lean mass and most have been conducted in adolescents or young adults.^14, 15, 16, 17, 18, 19, 20, 21, 22, 23^ This is despite the need to understand the factors that lead to reduced muscle mass and high fat mass in later adulthood when their health implications become clinically manifest. Previous studies have used limited measures of growth (typically spanning only infancy and childhood) and have focused exclusively on weight or BMI gain. Height gain may be also important for body composition, with recent findings in adolescents suggesting that weight and height gain may be independently associated with outcomes—greater weight gain in infancy was associated with higher fat mass in adolescence, whereas greater height gain in infancy was associated with lower fat mass.^21^ Most previous studies have also used relatively imprecise and inaccurate measures of body composition obtained by bioelectrical impedance or skin fold thickness^24, 25, 26^ and have only examined whole-body measures. Appendicular lean mass, which excludes bone and organ mass, is a more accurate indicator of skeletal muscle mass,^6, 27^ and measures of abdominal fat distribution may be more closely related to health than whole-body fat mass.^28^

The objectives of this study were to examine associations of birth weight, weight and height gain from infancy through to late adolescence with body composition in early old age using data from a large, nationally representative British birth cohort study—the Medical Research Council National Survey of Health and Development (NSHD). We hypothesised that birth weight would be positively associated with lean but not fat mass in early old age, and that weight gains in childhood and adolescence would be positively associated with both fat and lean mass.^29^ As taller individuals tend to have more fat and lean mass,^30^ we expected that measures of height gain would be positively associated with subsequent fat and lean mass due to the tracking of greater height and body size; and that these associations would be strongest when correlations between child and adult height were at their highest just before puberty and in late adolescence.^31^

## Materials and methods

### Study sample

The NSHD is a socially stratified sample of 5362 singleton births that took place in 1 week of March 1946 in mainland Britain,^32^ with regular follow-up across life. Between 2006 and 2010 (at 60–64 years), 2856 eligible study members (those known to be alive and with a known address in England, Scotland or Wales) were invited for an assessment at one of six clinical research facilities or to be visited by a research nurse at home.^33^ Seven hundred seventy-eight individuals had died and invitations were not sent to those who were living abroad (*n*=570), had previously withdrawn from the study (*n*=594) or who had been lost to follow-up (*n*=564). Of the 2856 invited, 2229 were assessed: 1690 attended a clinical research facility and the remaining 539 were seen at home.^34^

### Body composition measurement

During the visits to the clinical research facility, measures of body composition were obtained in the supine position using a QDR 4500 Discovery DXA scanner (Hologic Inc, Bedford, MA, USA); to optimise precision, scans were reviewed and centrally analysed (in Manchester) by a single operator (JA) using APEX 3.1 software (Hologic Inc., Bedford, MA, USA). Local quality assurance procedures were monitored centrally and cross-calibration between scanners was performed by scanning the European Spine Phantom at the start and end of the study.^35, 36^ From these scans, measures of fat (whole body, android and gynoid) and lean mass (whole body and appendicular) were obtained and converted into kilograms. Two ratios were derived: android/gynoid fat mass (higher values indicating greater fat distribution in the abdomen than hips) and whole body fat/lean mass; both of which were multiplied by 100. Lean mass was defined as mass excluding fat and bone mass, and in all measures data from the head were excluded due to the high proportion of bone mass known to affect the accuracy of soft-tissue measures.^37^ Routine anthropometric measures were taken during the clinic visit using standardised protocols by trained nurses.^33^ In total, 1558 of the 1690 eligible participants had data available for height, weight and body composition, with missing data largely due the presence of high-density artefacts in scans (*N*=74), technical or logistical problems (*n*=37), or participants laying outside the scan field (*n*=8).

### Measurement of weight and height

Birth weight, recorded to the nearest quarter of a pound, was extracted from birth records a few days after birth and converted into kilograms. Weight (kg) and height (cm) at 2, 4, 7, 11 and 15 years were measured by trained professionals using standardised protocols and self-reported at 20 years. Weight and height at each age were converted to sex-specific *z*-scores using the mean and standard deviation to aid comparisons of effect sizes at different ages.

### Analytical strategy

Linear regression models were used to examine associations of birth weight and weight and height from 2 to 20 years with each body composition outcome. The birth weight models were additionally adjusted for adult height at 60–64 years, considered a potential mediator. Weight and height at each age were mutually adjusted in order to estimate their independent associations.

To examine associations with periods of weight and height gain after birth, conditional growth models were constructed. Weight and height velocities (kg or cm year^−1^) were created using the exact age of measurement and converted into sex-specific *z*-scores. Separate models for each period (2–4, 4–7, 7–11, 11–15, 15–20 years) were constructed with weight and height velocity entered in the same model alongside weight and height *z*-score at the beginning of each period, using the maximum available sample in each period. Using weight as an example to highlight the interpretation of these models, the exposure is a standardised measure of weight change in a given interval on a theoretical distribution, which compares each individual against other cohort members with the same starting height and weight and the same height growth. As birth length was not measured, models examining weight gain from 0 to 2 years were adjusted for height at 2 years only. These conditional models were then further adjusted for adult height, a potential mediator. Analyses were conducted separately in males and females as sex differences in growth may underlie sexual dimorphism of adult body composition,^38^ with tests of sex interaction conducted to formally test these differences. Non-linearity was assessed in conditional models by the inclusion of a quadratic term (for weight or height velocity).

### Additional and sensitivity analyses

Additional analyses were conducted to examine whether associations were explained by socioeconomic position in childhood (paternal occupational class at 4 years), considered a potential confounder, and pubertal timing, a potential mediator (using prospectively ascertained examination of genitalia development and voice breaking status at 14 years (males) or age at menarche (females) as previously described).^39^ These models were also restricted to those with valid data in all periods to examine the potential influence of missing data on our findings. Last, to examine whether the mutual adjustment of weight and height impacted on findings, analyses were conducted in which weight gain was not adjusted for height at the beginning of the period or height gain velocity (and vice-versa).

## Results

### Sample description

The characteristics of the study sample at 60–64 years are shown in Table 1; the mean weights and heights at earlier ages are provided in Supplementary Table 1. As expected, sexual dimorphism of body composition was found. Despite both sexes having a similar mean BMI at 60–64 years, females had more fat and less lean mass (of the whole body and the limbs) than males, and males were on average heavier and taller, and had a higher android/gynoid ratio.

### Birth weight and body composition at 60–64 years

Higher birth weight was associated with higher whole body and appendicular lean mass and a lower android/gynoid ratio but was not associated with fat mass (Table 2). These associations with birth weight remained after adjustment for adult height except for the android/gynoid ratio in females. The associations of birth weight with whole body and appendicular lean mass were stronger in males than females (*P*-value for sex interaction term=0.01 in both cases).

### Weight and height from 2 to 20 years and body composition at 60–64 years

Associations between height-adjusted weight and weight-adjusted height at each age from 2 to 20 years and each outcome are shown in Table 3. Higher weight from 2 to 20 years was associated with higher fat mass and whole body and appendicular lean mass, whereas higher weight from 11 to 20 years was associated with higher fat/lean and android/gynoid ratios. Height was generally not associated with fat mass, except for inverse associations at 15 years in males. Taller height at each age was associated with higher whole body and appendicular lean mass, and was associated with lower fat/lean and android/gynoid ratios at most ages in men and from 11 years onwards in women.

### Conditional weight and height growth and body composition at 60–64 years

**Figure 1:**
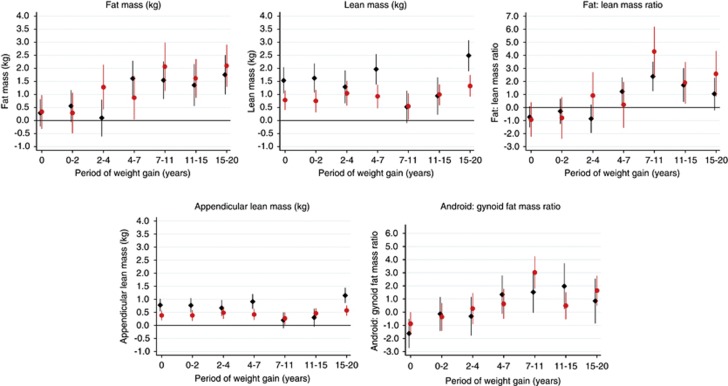
Mean differences in body composition outcomes at age 60–64 years (with 95% confidence intervals) per standard deviation increase in birth weight and weight gain velocity (adjusted for weight and height at the beginning of each period and concurrent height gain velocity). Notes: males=black diamonds; females=gray/red circles; sample sizes in the different periods were (male/female): birth weight (745/808); 0–2 (592/625); 2–4 (561/580); 4–7 (574/615); 7–11 (573/622); 11–15 (543/588); 15–20 (500/555); associations with weight gain from 0 to 2 years were adjusted for height at 2 years only. The full colour version of this figure is available at *International Journal of Obesity* online.

Figure 1 shows the associations between weight gain (adjusted for height gain in each respective interval and height and weight at the beginning of that interval) and each outcome (tabulated in Supplementary Table 2 model a). Greater weight gains across growth intervals from 4 to 20 years in males and from 2 to 20 years in females were associated with higher fat mass at age 60–64 years. Greater weight gains across growth intervals from 0 to 20 years were associated with higher whole body and appendicular lean mass. In both sexes, associations were weaker between 7 and 15 years than in other periods, whereas associations were stronger in males than in females from 0 to 2, 4 to 7 and 15 to 20 years. Greater weight gain in later childhood and adolescence (7–20 years) was associated with higher fat/lean mass and android/gynoid ratios. Similar overall patterns of association were found after adjustment for adult height (Supplementary Table 2 model b); for a given adult height, positive associations of early weight gain (0–4 years) with lean mass were partly attenuated, whereas positive associations with weight gain between 7 and 15 years in men and 7–11 years in women were strengthened.

**Figure 2:**
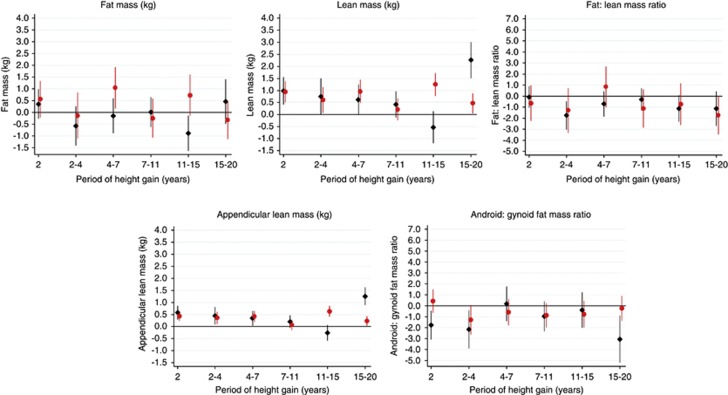
Mean differences in body composition outcomes at age 60–64 years (with 95% confidence intervals) per standard deviation increase in height at age 2 and height gain velocity (adjusted for height and weight at the beginning of each period and concurrent weight gain velocity). Notes: males=black diamonds; females=gray/red circles; sample sizes in the different periods were (male/female): 2 (593/627); 2–4 (561/580); 4–7 (574/615); 7–11 (573/622); 11–15 (543/588); 15–20 (500/555); associations with height at 2 years were adjusted for weight at 2 years only. The full colour version of this figure is available at *International Journal of Obesity* online.

Figure 2 shows the associations between height gain (adjusted for weight gain in each respective interval and height and weight at the beginning of that interval) and each outcome (tabulated in Supplementary Table 3 model a). Across growth intervals, height gain was generally not associated with fat mass. Height gain across growth intervals was more consistently positively associated with lean mass, except at 11–15 years in males; the positive associations were strongest in males at 15–20 years and strongest in females at 11–15 years. There was a weak negative association in males from 11 to 15 years. Early height gain (up to age 4 years) and height gain from 15 to 20 years (in males) was associated with a lower android/gynoid ratio and height gain from 2 to 4 and 15 to 20 years was weakly associated with a lower fat/lean mass ratio; otherwise greater height gain was not associated with these ratios. The associations between height gain and all these outcomes were generally not maintained after adjustment for adult height (Supplementary Table 3 model b).

There was little evidence for departure from linearity in any of the models.

### Additional and sensitivity analyses

Patterns of associations between conditional weight and height gain with each outcome were similar when additional adjustments were made for paternal occupational class and pubertal timing, and when restricted to a sample with valid growth data in all periods (Supplementary Tables 4 and 5 model a). Patterns of associations between conditional weight gain and outcomes were similar when no adjustment was made for baseline height and concurrent height gain (Supplementary Table 4 model b). However, associations between conditional height gain and outcomes differed where no adjustment was made for baseline weight and concurrent weight gain: in most periods, greater height gain was associated with higher fat and lean mass (Supplementary Table 5 model b).

## Discussion

### Main findings

This study found that higher birth weight was associated with higher lean mass (more strongly in males than in females) and lower android/gynoid ratio at age 60–64 years. Greater weight gains in infancy and early childhood and in late adolescence were associated with higher lean mass, whereas greater gains in later childhood and adolescence were associated with higher fat mass, and higher fat/lean mass and android/gynoid ratios. These patterns remained after adjustment for adult height. Across most growth intervals, greater height gain was associated with higher lean mass and lower fat/lean and android/gynoid ratios; these associations were mainly working through final adult height.

### Comparison with previous studies

This study extends previous studies that have generally been in younger cohorts, by examining the whole growth trajectory (including late adolescence) and including appendicular lean mass and fat/lean and android/gynoid ratios as additional outcomes. Our finding of an association between higher birth weight and higher lean mass is consistent with reports from younger cohort studies.^14, 40^ Our study shows that these associations persist into early old age and are also seen for appendicular lean mass and lower android/gynoid ratios.

In contrast with this study, previous studies in younger cohorts have reported positive associations between weight or BMI gain in infancy with fat mass.^16, 21, 22^ Our finding that weight gain in infancy and early childhood was more strongly associated with lean than fat mass is consistent with the only other study in early old age (56–70 years), which found that infant BMI gain was associated with adult lean mass, whereas BMI gain in childhood was associated with adult fat and lean mass.^29^

Findings from this study are consistent with previous findings from the NSHD at earlier ages using related measures of adiposity. For example, birth weight was positively associated with BMI and inversely associated with waist/hip ratio (females only) at 43 years (after adjustment for contemporaneous BMI),^41^ and greater weight gain in infancy was associated with higher BMI at 43 years.^12^ Findings from the present study suggest that these associations with obesity may primarily reflect an influence of weight gain on lean and not fat mass. Our findings are also consistent with our previous findings showing benefits of prenatal and prepubertal growth on muscle strength and physical performance at 53 years.^42, 43, 44^

### Explanation of findings

As the number of muscle fibres in adulthood is thought to be largely determined by birth,^45, 46, 47^ positive associations between birth weight and lean mass may reflect the acquisition of greater numbers of muscle fibres in utero (hyperplasia), which then track into adulthood. Alternatively, those of higher birth weight may have gone on to acquire more lean mass after birth (via hypertrophy). Positive associations of birth weight and early weight gain (0–4 years) with lean mass were partly explained by adult height—such that those who gained more weight went on to become taller adults with longer bones and longer muscles of greater mass. The persistence of positive associations after adjustment suggests that greater birth weight and weight gain also led to higher lean mass through other mechanisms such as the development of greater muscle width (the number of adjacent muscle fibres and/or their thickness), and/or the number of muscle fibres per unit area of muscle.^45^

Weight gains in later childhood and adolescence were more strongly associated than weight gain in infancy with fat mass in early old age. Later childhood and adolescence may be periods in which fat mass accrual is greater than in infancy^38, 48, 49^ and/or where changes in weight are related to formation of physical activity and diet patterns, which track into adulthood^50, 51^ leading to subsequent gains in fat mass.

Higher birth weight was associated with lower android/gynoid ratios, whereas greater gains in weight in later childhood and adolescence were associated with higher android/gynoid and fat/lean ratios. These associations were driven by the differing strengths of associations with fat and lean mass, and with android and gynoid fat mass (shown in Supplementary Table 6).

Height gain was generally not associated with fat mass and more consistently positively associated with lean mass. This probably reflects the stronger correlation between adult height and lean mass as associations between height gain and all outcomes generally operated through adult height.

It is important to note that associations between growth and body composition in adulthood may differ in younger cohorts that have experienced higher rates of childhood obesity—in these cohorts weight gain in infancy may predominantly reflect gains in fat mass. In older cohorts, as shown in this study, associations between infant weight gain and fat mass are likely to be weaker because weight gain in infancy may predominantly reflect gains in lean mass. Older cohorts are, however, important resources for examining the influence of growth on health-related outcomes in adulthood; findings from this study can be used to help interpret findings from other existing adult cohort studies, most of which have examined associations with BMI and not direct measures of body composition.

Differences in pubertal maturation could feasibly explain many of the observed associations, as greater weight gain in infancy and early childhood has been associated with earlier pubertal maturation, which in turn has been associated with greater fat mass in females^52^ and greater lean mass in males.^53^ However, the associations between weight gain and body composition outcomes found in this study were not explained by pubertal timing, potentially suggesting that there may be direct effects of weight gain on subsequent fat and lean mass. The associations were also not explained by childhood socioeconomic position, a potential confounder.

### Strengths and limitations

A major strength of this study is the use of detailed measures of whole body and regional body fat and lean mass obtained using DXA. Prospectively ascertained measures of weight and height gain spanning infancy, childhood and adolescence were used, and analyses conducted enabled associations with different periods of growth to be compared, and the separate associations of weight and height gain to be examined. Prospective measures of potential confounders and mediators were available in this study.

Although the measures of growth used in this study were extensive, other measures may be more closely related to body composition outcomes. For example, birth weight is only a crude indicator of prenatal growth that may predominantly reflect growth in the third trimester of pregnancy,^54^ and evidence from the Dutch ‘hunger winter' study of 1944–1945 suggests that impaired growth in the first two trimesters of pregnancy, but not the third, is associated with high subsequent fat mass.^55, 56^ Weight gain in the first weeks or months of infancy has also been found to be particularly important for subsequent fat mass,^16, 57^ but only weight gain between 0 and 2 years was available for investigation in this study.

Although missing data may have introduced bias, similar patterns of associations were found when comparing analyses run on maximum available samples with those restricted to the sample with valid growth data in all periods, suggesting that this source of bias is unlikely to have a substantial impact on findings. In addition, further analyses showed there was little difference in weight and height across the periods used when comparing participants with and without full body composition data (data available on request).

### Implications

Findings from this study suggest that growth in early life may have lasting effects on fat and lean mass, and highlight the opportunities that early-life interventions may have in preventing high fat mass and low lean mass in early old age. Our results suggest that greater weight gain in early life (before birth, in infancy and early childhood) may be beneficial by leading to higher lean mass and lower android/gynoid ratios, whereas greater weight gain in later childhood and adolescence may be detrimental by leading to higher fat/lean and android/gynoid ratio. A higher fat/lean ratio has been associated with poorer physical functioning,^58^ and a higher android/gynoid ratio associated with worse glucose metabolism.^59^ Future studies are needed to examine the factors that promote healthy weight gain, which may be comprised of greater lean than fat mass acquisition.

Across growth intervals greater height gain tended to be associated with lower fat/lean and android/gynoid ratios. These results support the suggestion that concurrent height and weight gain may have contrasting effects on subsequent body composition outcomes,^21^ although given the lack of research in this area, our findings require replication.

## Conclusions

This study found characteristics of the growth trajectory from birth to late adolescence were associated with body composition measures in early old age. Findings suggest that greater early weight and height gain lead to greater lean mass, protecting against the detrimental effects of declines in muscle mass that occur in later life, and have additional benefits by leading to a lower android/gynoid ratio. In contrast, greater weight gain in later childhood and adolescence leads to higher fat/lean and android/gynoid ratios, which could have detrimental effects on health and physical performance in adulthood.

## Figures and Tables

**Table 1:** Characteristics of the study sample at age 60–64 years stratified by sex

	*Mean (s.d.)*		
	*Males (*n*=746)*	*Females (*n*=812)*	P*-value*^a^
Weight (kg)	85.27 (13.05)	72.34 (13.63)	<0.001
Height (cm)	175.29 (6.45)	162.17 (5.76)	<0.001
Body mass index (kgm^−2^)	27.74 (3.94)	27.51 (5.02)	0.31
Whole-body fat mass (kg)	23.79 (7.19)	29.00 (9.22)	<0.001
Android fat mass (kg)	2.51 (0.96)	2.33 (1.01)	<0.001
Gynoid fat mass (kg)	3.76 (1.01)	5.11 (1.46)	<0.001
Android/gynoid ratio	65.69 (15.35)	44.74 (12.36)	<0.001
Whole-body lean mass (kg)	53.69 (7.06)	37.26 (5.35)	<0.001
Appendicular lean mass (kg)	24.62 (3.40)	16.21 (2.54)	<0.001
Fat/lean ratio	44.09 (10.99)	77.21 (18.91)	<0.001

a Comparison of sexes using *t*-tests. Sample restricted to those with complete data on all body composition measures at age 60–64 years.

**Table 2:** Mean differences in body composition outcomes per standard deviation increase in birth weight

*Outcome*	*Males (*n*=745)*		*Females (*n*=808)*		P#
	*Unadjusted*	*Adjusted for adult height*	*Unadjusted*	*Adjusted for adult height*	
Fat mass (kg)	0.29(−0.22, 0.81), 0.26	−0.01(−0.54, 0.52), 0.97	0.33(−0.31, 0.97), 0.31	0.09(−0.56, 0.74), 0.79	0.93
Lean mass (kg)	1.54(1.04, 2.03), <0.01	0.62(0.17, 1.07), 0.01	0.78(0.41, 1.14), <0.01	0.29(−0.05, 0.63), 0.09	0.01
Appendicular lean mass (kg)	0.78(0.54, 1.02), <0.01	0.33(0.11, 0.54), <0.01	0.38(0.21, 0.55), <0.01	0.15(−0.01, 0.31), 0.07	0.01
Fat/lean ratio	−0.73(−1.52, 0.06), 0.07	−0.53(−1.35, 0.29), 0.21	−0.92(−2.23, 0.38), 0.17	−0.58(−1.92, 0.75), 0.39	0.81
Android/gynoid ratio	−1.62(−2.72, −0.52), <0.01	−1.22(−2.36, −0.09), 0.04	−0.87(−1.72, −0.02), 0.05	−0.68(−1.55, 0.19), 0.13	0.29

Notes: cells show *β* (95% confidence intervals), and *P*-values; *P*#, *P*-value for sex interaction term (tested in unadjusted model).

**Table 3:** Mean differences in body composition outcomes at age 60–64 years per standard deviation increase in weight and height from 2 to 20 years. Height and weight at each age are mutually adjusted

*Outcome and age (year) of weight and height*	*Weight*		*Height*	
	*Males*	*Females*	*Males*	*Females*
*Fat mass (kg)*				
* *2	0.61 (−0.01, 1.22), 0.05	0.31 (−0.46, 1.08), 0.44	0.35 (−−0.26, 0.97), 0.26	0.56 (−0.21, 1.33), 0.15
* *4	0.63 (−0.04, 1.30), 0.06	1.29 (0.41, 2.17), <0.01	−0.19 (−0.86, 0.48), 0.57	0.09 (−0.77, 0.95), 0.83
* *7	1.76 (0.93, 2.58), <0.01	1.19 (0.25, 2.13), 0.01	−0.60 (−1.42, 0.22), 0.15	0.85 (−0.10, 1.80), 0.08
* *11	2.44 (1.71, 3.17), <0.01	2.56 (1.64, 3.48), <0.01	−0.62 (−1.35, 0.12), 0.10	0.04 (−0.88, 0.95), 0.94
* *15	3.03 (2.18, 3.88), <0.01	2.88 (2.11, 3.65), <0.01	−1.29 (−2.14, −0.44), <0.01	0.58 (−0.19, 1.35), 0.14
* *20	3.12 (2.49, 3.75), <0.01	3.63 (2.91, 4.34), <0.01	−0.46 (−1.09, 0.18), 0.16	−0.34 (−1.05, 0.38), 0.35
				
*Lean mass (kg)*				
* *2	2.03 (1.46, 2.59), <0.01	0.91 (0.48, 1.34), <0.01	0.99 (0.42, 1.55), <0.01	0.94 (0.51, 1.37), <0.01
* *4	2.06 (1.45, 2.66), <0.01	1.53 (1.05, 2.01), <0.01	0.87 (0.26, 1.48), 0.01	0.72 (0.24, 1.19), <0.01
* *7	2.98 (2.28, 3.68), <0.01	1.50 (1.00, 2.01), <0.01	0.72 (0.03, 1.42), 0.04	1.23 (0.72, 1.74), <0.01
* *11	2.29 (1.64, 2.94), <0.01	1.42 (0.92, 1.93), <0.01	1.43 (0.78, 2.07), <0.01	1.20 (0.70, 1.70), <0.01
* *15	2.82 (2.06, 3.59), <0.01	1.86 (1.45, 2.27), <0.01	0.56 (−0.20, 1.33), 0.15	1.57 (1.16, 1.98), <0.01
* *20	3.78 (3.29, 4.28), <0.01	2.51 (2.14, 2.88), <0.01	1.50 (1.00, 2.00), <0.01	1.15 (0.79, 1.52), <0.01
				
*Appendicular lean mass (kg)*				
* *2	0.96 (0.69, 1.24), <0.01	0.46 (0.26, 0.66), <0.01	0.57 (0.30, 0.85), <0.01	0.44 (0.23, 0.64), <0.01
* *4	1.05 (0.76, 1.34), <0.01	0.72 (0.50, 0.95), <0.01	0.47 (0.18, 0.77), <0.01	0.40 (0.18, 0.62), <0.01
* *7	1.43 (1.09, 1.76), <0.01	0.72 (0.48, 0.95), <0.01	0.42 (0.09, 0.76), 0.01	0.61 (0.37, 0.85), <0.01
* *11	1.05 (0.75, 1.36), <0.01	0.68 (0.45, 0.92), <0.01	0.77 (0.46, 1.08), <0.01	0.58 (0.34, 0.82), <0.01
* *15	1.25 (0.87, 1.62), <0.01	0.88 (0.69, 1.07), <0.01	0.34 (−0.04, 0.71), 0.08	0.77 (0.58, 0.96), <0.01
* *20	1.68 (1.44, 1.93), <0.01	1.16 (0.99, 1.33), <0.01	0.86 (0.61, 1.10), <0.01	0.59 (0.42, 0.77), <0.01
				
*Fat/lean ratio*				
* *2	−0.52 (−1.48, 0.44), 0.29	−1.14 (−2.74, 0.45), 0.16	−0.09 (−1.04, 0.87), 0.86	−0.64 (−2.22, 0.95), 0.43
* *4	−0.46 (−1.48, 0.56), 0.37	−0.24 (−2.03, 1.56), 0.80	−1.11 (−2.13, −0.10), 0.03	−0.90 (−2.66, 0.87), 0.32
* *7	0.67 (−0.62, 1.95), 0.31	−0.40 (−2.38, 1.58), 0.69	−1.61 (−2.89, −0.33), 0.01	−0.09 (−2.09, 1.91), 0.93
* *11	2.38 (1.22, 3.53), <0.01	3.41 (1.48, 5.34), <0.01	−2.16 (−3.32, −1.01), <0.01	−2.27 (−4.19, −0.35), 0.02
* *15	3.07 (1.71, 4.43), <0.01	3.38 (1.72, 5.03), <0.01	−2.71 (−4.06, −1.35), <0.01	−1.71 (−3.37, −0.05), 0.04
* *20	2.50 (1.47, 3.52), <0.01	3.92 (2.38, 5.46), <0.01	−2.03 (−3.07, −0.99), <0.01	−3.12 (−4.66, −1.58), <0.01
				
*Android/gynoid ratio*				
* *2	−0.55 (−1.86, 0.76), 0.41	−0.74 (−1.80, 0.32), 0.17	−1.77 (−3.08, −0.46), 0.01	0.42 (−0.63, 1.48), 0.43
* *4	−0.33 (−1.75, 1.09), 0.65	−0.10 (−1.27, 1.06), 0.86	−1.96 (−3.37, −0.54), 0.01	−0.96 (−2.11, 0.19), 0.10
* *7	0.62 (−1.15, 2.40), 0.49	0.45 (−0.83, 1.73), 0.49	−1.58 (−3.34, 0.18), 0.08	−1.19 (−2.48, 0.10), 0.07
* *11	2.16 (0.58, 3.75), 0.01	2.35 (1.11, 3.59), <0.01	−2.64 (−4.23, −1.05), <0.01	−1.88 (−3.12, −0.65), <0.01
* *15	3.16 (1.29, 5.04), <0.01	1.81 (0.76, 2.87), <0.01	−2.58 (−4.46, −0.71), 0.01	−1.37 (−2.43, −0.31), 0.01
* *20	2.28 (0.84, 3.73), <0.01	2.33 (1.30, 3.36), <0.01	−2.81 (−4.27, −1.35), <0.01	−2.05 (−3.08, −1.02), <0.01

Notes: cells show *β* (95% confidence intervals), and *P*-values; sample sizes in the different periods were (male/female): 2 (593/627), 4 (653/700), 7 (624/679), 11 (628/679), 15 (582/623), 20 (601/682).
