# Supplementary material for: Birth weight and growth from infancy to late adolescence in relation to fat and lean mass in early old age: findings from the MRC National Survey of Health and Development
Source: Int J Obes (Lond). 2013 Jul 16;38(1):69–75. doi: 10.1038/ijo.2013.115 (PMC3884138; doi:10.1038/ijo.2013.115)
Supplement: Supplementary Tables [file ijo2013115x1.doc]

Supplementary Table 1. Descriptive statistics showing the mean (SD) weight and height in infancy, childhood and adolescence

|  |  | Weight (kg) |  |  | Height (cm) |  |  |
| --- | --- | --- | --- | --- | --- | --- | --- |
| Age (year) | N (M/F) | Males | Females | P^a^ | Males | Females | P^a^ |
| Birth | 745/808 | 3.44 (0.52) | 3.35 (0.46) | <0.001 | - | - | - |
| 2 | 593/627 | 13.22 (1.39) | 12.67 (1.47) | <0.001 | 86.08 (5.00) | 85.04 (4.31) | <0.001 |
| 4 | 653/700 | 17.54 (2.12) | 17.21 (2.10) | <0.001 | 103.62 (4.85) | 103.55 (4.88) | 0.77 |
| 7 | 624/679 | 23.08 (2.78) | 22.81 (3.12) | 0.11 | 120.64 (5.21) | 120.40 (5.39) | 0.40 |
| 11 | 628/679 | 34.36 (5.74) | 35.36 (6.83) | <0.001 | 141.18 (6.59) | 141.98 (6.81) | 0.03 |
| 15 | 582/623 | 51.88 (9.21) | 52.34 (8.08) | 0.35 | 162.55 (8.96) | 159.37 (5.94) | <0.001 |
| 20 | 601/682 | 70.41 (8.74) | 57.79 (8.22) | <0.001 | 177.31 (6.54) | 163.14 (6.05) | <0.001 |

^a^Comparison of sexes using t-tests.

Supplementary Table 2. Mean differences in body composition outcomes per standard deviation increase in weight gain velocity (adjusted for weight and height at the beginning of each period and concurrent height gain velocity) a) before and b) after adjustment for adult height

| Outcome and period  of weight gain (years) | Males  a) | b) | Females  a) | b) | P# |
| --- | --- | --- | --- | --- | --- |
| Fat mass (kg) |  |  |  |  |  |
| 0-2* | 0.55(-0.05, 1.16), 0.07 | 0.37(-0.25, 0.99), 0.24 | 0.29(-0.48, 1.06), 0.46 | 0.07(-0.71, 0.85), 0.85 | 0.60 |
| 2–4 | 0.10(-0.60, 0.79), 0.79 | -0.07(-0.76, 0.63), 0.85 | 1.28(0.43, 2.13), <0.01 | 1.21(0.36, 2.06), 0.01 | 0.04 |
| 4–7 | 1.61(0.93, 2.28), <0.01 | 1.63(0.96, 2.30), <0.01 | 0.87(0.05, 1.70), 0.04 | 0.95(0.12, 1.78), 0.02 | 0.18 |
| 7–11 | 1.55(0.83, 2.26), <0.01 | 1.74(1.02, 2.46), <0.01 | 2.06(1.15, 2.98), <0.01 | 2.59(1.62, 3.56), <0.01 | 0.39 |
| 11–15 | 1.36(0.56, 2.16), <0.01 | 1.79(0.97, 2.62), <0.01 | 1.61(0.87, 2.35), <0.01 | 1.63(0.89, 2.37), <0.01 | 0.66 |
| 15-20 | 1.76(1.02, 2.50), <0.01 | 1.75(1.01, 2.49), <0.01 | 2.10(1.30, 2.90), <0.01 | 2.14(1.33, 2.95), <0.01 | 0.55 |
| Lean mass (kg) |  |  |  |  |  |
| 0-2* | 1.63(1.08, 2.18), <0.01 | 0.93(0.42, 1.45), <0.01 | 0.75(0.32, 1.17), <0.01 | 0.30(-0.10, 0.70), 0.15 | 0.01 |
| 2–4 | 1.29(0.67, 1.91), <0.01 | 0.88(0.30, 1.46), <0.01 | 1.05(0.58, 1.51), <0.01 | 0.90(0.46, 1.34), <0.01 | 0.54 |
| 4–7 | 1.96(1.39, 2.54), <0.01 | 2.01(1.47, 2.55), <0.01 | 0.92(0.48, 1.37), <0.01 | 1.09(0.65, 1.52), <0.01 | <0.01 |
| 7–11 | 0.52(-0.09, 1.14), 0.10 | 0.95(0.36, 1.54), <0.01 | 0.54(0.05, 1.04), 0.03 | 1.28(0.77, 1.78), <0.01 | 0.96 |
| 11–15 | 0.94(0.24, 1.65), 0.01 | 1.98(1.31, 2.66), <0.01 | 0.99(0.59, 1.38), <0.01 | 1.11(0.73, 1.49), <0.01 | 0.91 |
| 15-20 | 2.49(1.91, 3.07), <0.01 | 2.43(1.85, 3.00), <0.01 | 1.33(0.92, 1.73), <0.01 | 1.24(0.84, 1.65), <0.01 | <0.01 |
| Appendicular lean mass (kg) |  |  |  |  |  |
| 0-2* | 0.77(0.50, 1.03), <0.01 | 0.42(0.18, 0.67), <0.01 | 0.38(0.18, 0.58), <0.01 | 0.17(-0.02, 0.36), 0.08 | 0.02 |
| 2–4 | 0.67(0.37, 0.97), <0.01 | 0.47(0.20, 0.75), <0.01 | 0.48(0.26, 0.70), <0.01 | 0.41(0.21, 0.62), <0.01 | 0.31 |
| 4–7 | 0.92(0.64, 1.20), <0.01 | 0.94(0.69, 1.20), <0.01 | 0.42(0.21, 0.63), <0.01 | 0.49(0.29, 0.69), <0.01 | <0.01 |
| 7–11 | 0.20(-0.09, 0.49), 0.18 | 0.41(0.13, 0.69), <0.01 | 0.26(0.03, 0.50), 0.03 | 0.61(0.38, 0.84), <0.01 | 0.72 |
| 11–15 | 0.30(-0.04, 0.64), 0.09 | 0.82(0.50, 1.15), <0.01 | 0.46(0.28, 0.65), <0.01 | 0.52(0.34, 0.70), <0.01 | 0.38 |
| 15-20 | 1.15(0.87, 1.43), <0.01 | 1.12(0.84, 1.39), <0.01 | 0.57(0.38, 0.76), <0.01 | 0.54(0.35, 0.73), <0.01 | <0.01 |
| Fat: lean ratio |  |  |  |  |  |
| 0-2* | -0.29(-1.23, 0.65), 0.55 | -0.04(-1.01, 0.92), 0.93 | -0.80(-2.38, 0.78), 0.32 | -0.49(-2.10, 1.13), 0.55 | 0.59 |
| 2–4 | -0.87(-1.94, 0.20), 0.11 | -0.83(-1.91, 0.25), 0.13 | 0.92(-0.86, 2.69), 0.31 | 1.01(-0.76, 2.79), 0.26 | 0.09 |
| 4–7 | 1.23(0.18, 2.29), 0.02 | 1.23(0.18, 2.29), 0.02 | 0.20(-1.53, 1.93), 0.82 | 0.09(-1.65, 1.83), 0.92 | 0.33 |
| 7–11 | 2.38(1.26, 3.49), <0.01 | 2.37(1.24, 3.51), <0.01 | 4.28(2.37, 6.19), <0.01 | 4.22(2.18, 6.26), <0.01 | 0.10 |
| 11–15 | 1.72(0.44, 3.00), 0.01 | 1.62(0.28, 2.95), 0.02 | 1.89(0.32, 3.47), 0.02 | 1.72(0.14, 3.29), 0.03 | 0.88 |
| 15-20 | 1.04(-0.18, 2.27), 0.10 | 1.09(-0.14, 2.31), 0.08 | 2.59(0.86, 4.32), <0.01 | 2.82(1.08, 4.55), <0.01 | 0.17 |
| Android: gynoid ratio |  |  |  |  |  |
| 0-2* | -0.14(-1.42, 1.15), 0.84 | 0.20(-1.11, 1.52), 0.76 | -0.36(-1.41, 0.69), 0.50 | -0.15(-1.22, 0.92), 0.79 | 0.79 |
| 2–4 | -0.31(-1.77, 1.15), 0.68 | -0.18(-1.65, 1.30), 0.81 | 0.27(-0.90, 1.44), 0.65 | 0.30(-0.88, 1.47), 0.62 | 0.54 |
| 4–7 | 1.34(-0.11, 2.79), 0.07 | 1.31(-0.13, 2.76), 0.08 | 0.64(-0.48, 1.75), 0.26 | 0.65(-0.48, 1.77), 0.26 | 0.45 |
| 7–11 | 1.52(-0.03, 3.08), 0.05 | 1.17(-0.40, 2.73), 0.15 | 3.02(1.80, 4.23), <0.01 | 3.32(2.03, 4.62), <0.01 | 0.14 |
| 11–15 | 1.98(0.25, 3.71), 0.03 | 1.44(-0.36, 3.25), 0.12 | 0.49(-0.52, 1.50), 0.34 | 0.57(-0.45, 1.58), 0.27 | 0.13 |
| 15-20 | 0.85(-0.84, 2.54), 0.32 | 0.90(-0.79, 2.59), 0.30 | 1.64(0.51, 2.76), <0.01 | 1.56(0.43, 2.69), 0.01 | 0.44 |

Notes: cells show β (95% confidence intervals), and p-values; Sample sizes in the different periods were (male/female): 0–2 (592/625); 2–4 (561/580); 4–7 (574/615); 7–11 (573/622); 11–15 (543/588); 15–20 (500/555)

*adjusted for height at 2 years

P# p-value for sex interaction term in model a.

Supplementary Table 3. Mean differences in body composition outcomes per standard deviation increase in height at age 2 and height gain velocity (adjusted for height and weight at the beginning of each period and concurrent weight gain velocity) a) before and b) after adjustment for adult height

| Outcome and period  of height gain (years) | Males  a) | b) | Females  a) | b) | P# |
| --- | --- | --- | --- | --- | --- |
| Fat mass (kg) |  |  |  |  |  |
| 2* | 0.35(-0.26, 0.97), 0.26 | 0.08(-0.57, 0.73), 0.81 | 0.56(-0.21, 1.33), 0.15 | 0.24(-0.56, 1.05), 0.55 | 0.69 |
| 2–4 | -0.58(-1.41, 0.24), 0.17 | -1.19(-2.08, -0.29), 0.01 | -0.13(-1.10, 0.83), 0.78 | -0.64(-1.71, 0.44), 0.24 | 0.50 |
| 4–7 | -0.15(-0.87, 0.58), 0.69 | -0.69(-1.47, 0.09), 0.08 | 1.05(0.18, 1.92), 0.02 | 0.67(-0.29, 1.63), 0.17 | 0.04 |
| 7–11 | 0.02(-0.61, 0.64), 0.96 | -0.40(-1.08, 0.27), 0.24 | -0.25(-1.07, 0.57), 0.55 | -0.65(-1.50, 0.21), 0.14 | 0.61 |
| 11–15 | -0.89(-1.63, -0.15), 0.02 | -1.17(-1.92, -0.42), <0.01 | 0.73(-0.14, 1.60), 0.10 | 0.50(-0.70, 1.70), 0.41 | 0.01 |
| 15-20 | 0.46(-0.48, 1.40), 0.33 | 0.37(-1.19, 1.93), 0.64 | -0.32(-1.12, 0.48), 0.43 | 0.00(-1.08, 1.08), 1.00 | 0.24 |
| Lean mass (kg) |  |  |  |  |  |
| 2* | 0.99(0.42, 1.55), <0.01 | -0.11(-0.65, 0.43), 0.70 | 0.94(0.51, 1.37), <0.01 | 0.26(-0.16, 0.68), 0.22 | 0.90 |
| 2–4 | 0.76(0.02, 1.49), 0.05 | -0.76(-1.50, -0.01), 0.05 | 0.61(0.08, 1.13), 0.02 | -0.42(-0.97, 0.14), 0.14 | 0.74 |
| 4–7 | 0.62(0.00, 1.24), 0.05 | -0.48(-1.11, 0.14), 0.13 | 0.96(0.49, 1.43), <0.01 | 0.19(-0.31, 0.69), 0.45 | 0.38 |
| 7–11 | 0.42(-0.11, 0.96), 0.12 | -0.48(-1.04, 0.07), 0.09 | 0.22(-0.22, 0.66), 0.33 | -0.34(-0.78, 0.11), 0.14 | 0.56 |
| 11–15 | -0.53(-1.18, 0.13), 0.11 | -1.20(-1.82, -0.59), <0.01 | 1.25(0.78, 1.71), <0.01 | -0.11(-0.73, 0.52), 0.74 | <0.01 |
| 15-20 | 2.26(1.53, 3.00), <0.01 | 0.12(-1.08, 1.32), 0.84 | 0.47(0.06, 0.87), 0.03 | -0.24(-0.78, 0.31), 0.39 | <0.01 |
| Appendicular lean mass (kg) |  |  |  |  |  |
| 2* | 0.57(0.30, 0.85), <0.01 | 0.03(-0.23, 0.29), 0.81 | 0.44(0.23, 0.64), <0.01 | 0.12(-0.08, 0.32), 0.24 | 0.42 |
| 2–4 | 0.44(0.09, 0.80), 0.01 | -0.29(-0.64, 0.07), 0.12 | 0.36(0.11, 0.60), <0.01 | -0.09(-0.35, 0.17), 0.49 | 0.69 |
| 4–7 | 0.34(0.05, 0.64), 0.02 | -0.20(-0.50, 0.10), 0.20 | 0.42(0.20, 0.64), <0.01 | 0.07(-0.16, 0.31), 0.55 | 0.67 |
| 7–11 | 0.21(-0.05, 0.46), 0.11 | -0.25(-0.51, 0.01), 0.06 | 0.07(-0.14, 0.27), 0.53 | -0.20(-0.40, 0.01), 0.06 | 0.40 |
| 11–15 | -0.26(-0.58, 0.05), 0.10 | -0.60(-0.90, -0.31), <0.01 | 0.64(0.42, 0.85), <0.01 | 0.05(-0.25, 0.34), 0.76 | <0.01 |
| 15-20 | 1.25(0.89, 1.61), <0.01 | 0.04(-0.55, 0.62), 0.90 | 0.23(0.04, 0.42), 0.03 | -0.05(-0.31, 0.20), 0.39 | <0.01 |
| Fat: lean ratio |  |  |  |  |  |
| 2* | -0.09(-1.04, 0.87), 0.86 | 0.31(-0.70, 1.33), 0.55 | -0.64(-2.22, 0.95), 0.43 | -0.11(-1.78, 1.56), 0.90 | 0.57 |
| 2–4 | -1.75(-3.01, -0.48), 0.01 | -1.60(-2.99, -0.22), 0.02 | -1.29(-3.30, 0.72), 0.21 | -0.62(-2.85, 1.62), 0.59 | 0.71 |
| 4–7 | -0.71(-1.85, 0.43), 0.22 | -0.78(-2.01, 0.45), 0.22 | 0.84(-0.98, 2.67), 0.37 | 1.36(-0.66, 3.38), 0.19 | 0.17 |
| 7–11 | -0.30(-1.28, 0.67), 0.54 | -0.29(-1.36, 0.77), 0.59 | -1.14(-2.84, 0.57), 0.19 | -1.09(-2.88, 0.71), 0.24 | 0.41 |
| 11–15 | -1.12(-2.30, 0.07), 0.07 | -1.05(-2.26, 0.16), 0.09 | -0.73(-2.59, 1.13), 0.44 | 1.21(-1.34, 3.76), 0.35 | 0.74 |
| 15-20 | -1.14(-2.69, 0.42), 0.15 | 0.53(-2.04, 3.10), 0.69 | -1.73(-3.46, <0.01), 0.05 | 0.15(-2.17, 2.47), 0.90 | 0.65 |
| Android: gynoid ratio |  |  |  |  |  |
| 2* | -1.77(-3.08, -0.46), 0.01 | -1.17(-2.56, 0.21), 0.10 | 0.42(-0.63, 1.48), 0.43 | 0.80(-0.31, 1.91), 0.16 | 0.01 |
| 2–4 | -2.16(-3.89, -0.43), 0.01 | -1.67(-3.56, 0.22), 0.08 | -1.29(-2.61, 0.04), 0.06 | -1.11(-2.58, 0.37), 0.14 | 0.43 |
| 4–7 | 0.18(-1.39, 1.74), 0.83 | 0.83(-0.86, 2.51), 0.34 | -0.59(-1.77, 0.59), 0.32 | -0.64(-1.94, 0.67), 0.34 | 0.44 |
| 7–11 | -0.96(-2.32, 0.39), 0.16 | -0.20(-1.67, 1.27), 0.79 | -0.87(-1.96, 0.22), 0.12 | -1.10(-2.24, 0.04), 0.06 | 0.92 |
| 11–15 | -0.39(-2.00, 1.22), 0.63 | -0.04(-1.68, 1.60), 0.96 | -0.77(-1.96, 0.43), 0.21 | -1.63(-3.27, 0.01), 0.05 | 0.71 |
| 15-20 | -3.06(-5.20, -0.92), 0.01 | -1.28(-4.83, 2.28), 0.48 | -0.23(-1.35, 0.89), 0.68 | -0.84(-2.36, 0.67), 0.27 | 0.02 |

Notes: cells show β (95% confidence intervals), and p-values; sample sizes in the different periods were (male/female): 2 (593/627); 2–4 (561/580); 4–7 (574/615); 7–11 (573/622); 11–15 (543/588); 15–20 (500/555)

*adjusted for weight at 2 years

P# p-value for sex interaction term in model a.

Supplementary Table 4. Mean differences in body composition outcomes per standard deviation increase in birth weight and weight gain velocity, a) adjusted for weight and height at the beginning of each period, concurrent height gain velocity, father’s occupational class at 4 years and pubertal timing; and b) adjusted for weight at the beginning of each period

| Outcome and period  of weight gain (years) | Males  a) | b) | Females  a) | b) |
| --- | --- | --- | --- | --- |
| Fat mass (kg) |  |  |  |  |
| Birth weight | -0.09(-0.89, 0.71), 0.83 | 0.29(-0.22, 0.81), 0.26 | 0.85(-0.11, 1.81), 0.08 | 0.33(-0.31, 0.97), 0.31 |
| 0-2* | 0.54(-0.29, 1.38), 0.21 | 0.64(0.06, 1.23), 0.03 | 0.41(-0.65, 1.47), 0.45 | 0.49(-0.23, 1.20), 0.18 |
| 2–4 | 0.40(-0.60, 1.40), 0.43 | 0.05(-0.55, 0.66), 0.86 | 0.77(-0.36, 1.91), 0.18 | 1.29(0.54, 2.03), <0.01 |
| 4–7 | 2.14(1.19, 3.08), <0.01 | 1.33(0.75, 1.90), <0.01 | 0.64(-0.51, 1.79), 0.28 | 1.27(0.54, 1.99), <0.01 |
| 7–11 | 2.31(1.32, 3.29), <0.01 | 1.50(0.83, 2.18), <0.01 | 1.46(0.13, 2.79), 0.03 | 1.94(1.13, 2.74), <0.01 |
| 11–15 | 2.06(0.97, 3.15), <0.01 | 0.60(-0.02, 1.22), 0.06 | 0.99(-0.06, 2.05), 0.07 | 1.82(1.13, 2.52), <0.01 |
| 15-20 | 2.05(1.15, 2.95), <0.01 | 1.96(1.31, 2.60), <0.01 | 2.65(1.63, 3.67), <0.01 | 2.04(1.28, 2.79), <0.01 |
| Lean mass (kg) |  |  |  |  |
| Birth weight | 1.36(0.60, 2.11), <0.01 | 1.54(1.04, 2.03), <0.01 | 1.24(0.67, 1.81), <0.01 | 0.78(0.41, 1.14), <0.01 |
| 0-2* | 1.57(0.81, 2.32), <0.01 | 1.86(1.33, 2.40), <0.01 | 0.58(-0.03, 1.19), 0.07 | 1.08(0.67, 1.48), <0.01 |
| 2–4 | 1.67(0.79, 2.55), <0.01 | 1.70(1.16, 2.24), <0.01 | 1.01(0.37, 1.64), <0.01 | 1.42(1.01, 1.83), <0.01 |
| 4–7 | 2.00(1.20, 2.81), <0.01 | 2.20(1.71, 2.69), <0.01 | 0.67(0.04, 1.31), 0.04 | 1.38(0.98, 1.77), <0.01 |
| 7–11 | 1.03(0.18, 1.89), 0.02 | 0.73(0.15, 1.31), 0.01 | 0.70(-0.02, 1.42), 0.06 | 0.67(0.23, 1.11), <0.01 |
| 11–15 | 1.50(0.53, 2.48), <0.01 | 0.78(0.23, 1.33), 0.01 | 0.80(0.24, 1.35), 0.01 | 1.35(0.96, 1.73), <0.01 |
| 15-20 | 2.39(1.67, 3.11), <0.01 | 3.37(2.85, 3.89), <0.01 | 1.32(0.79, 1.85), <0.01 | 1.66(1.26, 2.06), <0.01 |
| Appendicular lean mass (kg) |  |  |  |  |
| Birth weight | 0.71(0.35, 1.07), <0.01 | 0.78(0.54, 1.02), <0.01 | 0.62(0.35, 0.88), <0.01 | 0.38(0.21, 0.55), <0.01 |
| 0-2* | 0.71(0.34, 1.07), <0.01 | 0.91(0.65, 1.16), <0.01 | 0.32(0.03, 0.60), 0.03 | 0.53(0.34, 0.73), <0.01 |
| 2–4 | 0.88(0.46, 1.30), <0.01 | 0.91(0.65, 1.17), <0.01 | 0.50(0.20, 0.80), <0.01 | 0.68(0.49, 0.87), <0.01 |
| 4–7 | 0.93(0.55, 1.31), <0.01 | 1.07(0.84, 1.31), <0.01 | 0.33(0.03, 0.63), 0.03 | 0.63(0.45, 0.82), <0.01 |
| 7–11 | 0.44(0.03, 0.85), 0.03 | 0.31(0.03, 0.58), 0.03 | 0.31(-0.03, 0.65), 0.07 | 0.30(0.10, 0.51), <0.01 |
| 11–15 | 0.59(0.12, 1.05), 0.01 | 0.24(-0.03, 0.51), 0.08 | 0.39(0.13, 0.65), <0.01 | 0.65(0.47, 0.83), <0.01 |
| 15-20 | 1.01(0.66, 1.37), <0.01 | 1.64(1.38, 1.90), <0.01 | 0.57(0.32, 0.82), <0.01 | 0.74(0.55, 0.92), <0.01 |
| Fat: lean ratio |  |  |  |  |
| Birth weight | -1.32(-2.54, -0.10), 0.04 | -0.73(-1.52, 0.06), 0.07 | -0.65(-2.57, 1.28), 0.51 | -0.92(-2.23, 0.38), 0.17 |
| 0-2* | -0.31(-1.59, 0.97), 0.63 | -0.30(-1.20, 0.61), 0.52 | -0.22(-2.37, 1.92), 0.84 | -1.02(-2.50, 0.46), 0.18 |
| 2–4 | -0.69(-2.21, 0.84), 0.38 | -1.28(-2.21, -0.35), 0.01 | -0.48(-2.78, 1.82), 0.68 | 0.26(-1.28, 1.81), 0.74 |
| 4–7 | 2.10(0.63, 3.56), 0.01 | 0.55(-0.35, 1.45), 0.23 | 0.30(-2.08, 2.68), 0.80 | 0.40(-1.11, 1.91), 0.60 |
| 7–11 | 3.46(1.92, 5.00), <0.01 | 2.14(1.09, 3.19), <0.01 | 2.37(-0.39, 5.13), 0.09 | 3.68(1.99, 5.36), <0.01 |
| 11–15 | 2.43(0.70, 4.17), 0.01 | 0.52(-0.49, 1.53), 0.31 | 0.63(-1.60, 2.86), 0.58 | 1.69(0.21, 3.16), 0.03 |
| 15-20 | 1.63(0.14, 3.13), 0.03 | 0.63(-0.45, 1.71), 0.25 | 4.11(1.91, 6.31), <0.01 | 1.77(0.14, 3.41), 0.03 |
| Android: gynoid ratio |  |  |  |  |
| Birth weight | -2.08(-3.70, -0.45), 0.01 | -1.62(-2.72, -0.52), <0.01 | 0.08(-1.20, 1.35), 0.91 | -0.87(-1.72, -0.02), 0.05 |
| 0-2* | 0.48(-1.21, 2.18), 0.58 | -0.56(-1.80, 0.68), 0.37 | -0.41(-1.82, 1.01), 0.58 | -0.21(-1.19, 0.76), 0.67 |
| 2–4 | 0.13(-1.90, 2.16), 0.90 | -1.42(-2.69, -0.15), 0.03 | 0.48(-1.03, 1.99), 0.54 | -0.21(-1.23, 0.81), 0.69 |
| 4–7 | 1.76(-0.20, 3.71), 0.08 | 0.77(-0.47, 2.01), 0.22 | -0.38(-1.95, 1.19), 0.63 | 0.14(-0.84, 1.12), 0.78 |
| 7–11 | 3.22(1.12, 5.32), <0.01 | 1.03(-0.43, 2.49), 0.17 | 2.94(1.14, 4.75), <0.01 | 2.55(1.47, 3.62), <0.01 |
| 11–15 | 2.05(-0.28, 4.39), 0.09 | 1.17(-0.19, 2.53), 0.09 | 0.31(-1.15, 1.77), 0.68 | 0.27(-0.68, 1.22), 0.58 |
| 15-20 | 1.69(-0.33, 3.70), 0.10 | -0.32(-1.80, 1.16), 0.67 | 1.55(0.08, 3.01), 0.04 | 1.34(0.28, 2.40), 0.01 |

Notes: cells show β (95% confidence intervals), and p-values; sample sizes in the different periods were (male/female), model a: 334 males and 354 females in all periods; model b: birth weight (745/808); 0–2 (592/625); 2–4 (561/580); 4–7 (574/615); 7–11 (573/622); 11–15 (543/588); 15–20 (500/555)

*adjusted for height at 2 years

Pubertal timing was categorised in four groups using prospectively ascertained physical examinations of genitalia development and voice breaking status at 14 years (males) or age at menarche (females) as previously described.^37^ Father’s occupational class (Registrar General’s social classification) was also included as a categorical term, from 1(professional) to 6(unskilled) and was prospectively ascertained at 4 years, or 11(n=5) or 15(n=3) if unavailable then.

Supplementary Table 5. Mean differences in body composition outcomes per standard deviation increase in height at 2 years and height gain velocity, a) adjusted for height and weight at the beginning of each period, concurrent weight gain velocity, father’s occupational class at 4 years and pubertal timing; and b) adjusted for height at the beginning of each period

| Outcome and period  of height gain (years) | Males  a) | b) | Females  a) | b) |
| --- | --- | --- | --- | --- |
| Fat mass (kg) |  |  |  |  |
| 2* | 0.24(-0.60, 1.08), 0.57 | 0.55(-0.01, 1.12), 0.06 | 1.06(-0.01, 2.13), 0.05 | 0.60(-0.09, 1.30), 0.09 |
| 2–4 | -0.66(-1.73, 0.41), 0.23 | -0.24(-0.95, 0.48), 0.51 | 0.21(-1.03, 1.45), 0.74 | 0.64(-0.18, 1.46), 0.13 |
| 4–7 | -0.34(-1.35, 0.66), 0.51 | 0.84(0.23, 1.45), 0.01 | 1.68(0.52, 2.83), <0.01 | 1.63(0.89, 2.38), <0.01 |
| 7–11 | -0.38(-1.22, 0.46), 0.38 | 0.73(0.14, 1.31), 0.02 | 0.35(-0.79, 1.50), 0.54 | 0.84(0.12, 1.55), 0.02 |
| 11–15 | -0.28(-1.34, 0.78), 0.61 | 0.03(-0.58, 0.65), 0.92 | 1.13(-0.11, 2.37), 0.07 | 1.10(0.27, 1.93), 0.01 |
| 15-20 | 0.02(-1.21, 1.25), 0.98 | 1.07(0.20, 1.94), 0.02 | 0.34(-0.71, 1.39), 0.53 | -0.20(-0.99, 0.60), 0.63 |
| Lean mass (kg) |  |  |  |  |
| 2* | 1.16(0.40, 1.92), <0.01 | 1.63(1.09, 2.17), <0.01 | 1.34(0.72, 1.96), <0.01 | 1.21(0.82, 1.61), <0.01 |
| 2–4 | 0.58(-0.36, 1.52), 0.23 | 1.94(1.29, 2.60), <0.01 | 0.81(0.11, 1.51), 0.02 | 1.36(0.90, 1.81), <0.01 |
| 4–7 | 0.64(-0.23, 1.50), 0.15 | 2.11(1.57, 2.65), <0.01 | 1.30(0.66, 1.93), <0.01 | 1.65(1.24, 2.06), <0.01 |
| 7–11 | 0.05(-0.67, 0.78), 0.89 | 1.02(0.50, 1.53), <0.01 | 0.85(0.23, 1.47), 0.01 | 0.70(0.32, 1.09), <0.01 |
| 11–15 | -0.20(-1.15, 0.75), 0.68 | 0.14(-0.40, 0.68), 0.62 | 1.37(0.73, 2.02), <0.01 | 1.46(1.01, 1.91), <0.01 |
| 15-20 | 2.06(1.07, 3.05), <0.01 | 3.32(2.58, 4.06), <0.01 | 0.51(-0.04, 1.06), 0.07 | 0.51(0.09, 0.93), 0.02 |
| Appendicular lean mass (kg) |  |  |  |  |
| 2* | 0.73(0.36, 1.09), <0.01 | 0.88(0.62, 1.14), <0.01 | 0.61(0.32, 0.90), <0.01 | 0.58(0.39, 0.76), <0.01 |
| 2–4 | 0.36(-0.09, 0.81), 0.12 | 1.03(0.71, 1.34), <0.01 | 0.38(0.05, 0.70), 0.02 | 0.71(0.50, 0.92), <0.01 |
| 4–7 | 0.44(0.03, 0.85), 0.04 | 1.06(0.80, 1.32), <0.01 | 0.55(0.25, 0.85), <0.01 | 0.75(0.56, 0.94), <0.01 |
| 7–11 | 0.05(-0.30, 0.39), 0.79 | 0.47(0.23, 0.72), <0.01 | 0.33(0.04, 0.62), 0.03 | 0.30(0.12, 0.49), <0.01 |
| 11–15 | -0.16(-0.61, 0.30), 0.50 | -0.03(-0.29, 0.23), 0.80 | 0.65(0.34, 0.95), <0.01 | 0.73(0.52, 0.94), <0.01 |
| 15-20 | 1.19(0.70, 1.67), <0.01 | 1.75(1.39, 2.10), <0.01 | 0.28(0.03, 0.54), 0.07 | 0.23(0.04, 0.43), 0.02 |
| Fat: lean ratio |  |  |  |  |
| 2* | -0.49(-1.78, 0.79), 0.45 | -0.25(-1.13, 0.63), 0.58 | -0.06(-2.22, 2.09), 0.95 | -1.10(-2.54, 0.35), 0.14 |
| 2–4 | -1.74(-3.37, -0.12), 0.04 | -2.07(-3.16, -0.97), <0.01 | -0.70(-3.22, 1.81), 0.58 | -1.00(-2.69, 0.69), 0.25 |
| 4–7 | -1.09(-2.65, 0.47), 0.17 | -0.17(-1.11, 0.76), 0.72 | 1.64(-0.74, 4.01), 0.18 | 0.75(-0.80, 2.30), 0.34 |
| 7–11 | -0.73(-2.04, 0.58), 0.27 | 0.48(-0.42, 1.39), 0.30 | -0.96(-3.33, 1.40), 0.43 | 0.64(-0.84, 2.13), 0.40 |
| 11–15 | -0.18(-1.87, 1.52), 0.84 | 0.01(-0.95, 0.97), 0.99 | 0.06(-2.55, 2.67), 0.96 | -0.28(-2.02, 1.45), 0.75 |
| 15-20 | -1.76(-3.80, 0.28), 0.09 | -0.94(-2.30, 0.42), 0.17 | -0.09(-2.36, 2.17), 0.94 | -1.47(-3.11, 0.16), 0.08 |
| Android: gynoid ratio |  |  |  |  |
| 2* | -1.65(-3.36, 0.06), 0.06 | -1.90(-3.11, -0.69), <0.01 | 0.46(-0.96, 1.89), 0.53 | 0.08(-0.87, 1.04), 0.86 |
| 2–4 | -2.58(-4.75, -0.42), 0.02 | -2.30(-3.79, -0.80), <0.01 | -1.29(-2.95, 0.36), 0.13 | -1.20(-2.31, -0.09), 0.03 |
| 4–7 | 0.41(-1.69, 2.50), 0.70 | 0.71(-0.58, 1.99), 0.28 | 1.22(-0.35, 2.79), 0.13 | -0.30(-1.30, 0.70), 0.56 |
| 7–11 | -2.38(-4.16, -0.60), 0.01 | -0.38(-1.63, 0.87), 0.55 | -0.63(-2.17, 0.92), 0.43 | 0.44(-0.51, 1.38), 0.37 |
| 11–15 | 1.07(-1.21, 3.35), 0.36 | 0.87(-0.41, 2.16), 0.18 | -1.41(-3.12, 0.30), 0.11 | -0.81(-1.92, 0.30), 0.15 |
| 15-20 | -3.76(-6.51, -1.01), 0.01 | -2.93(-4.78, -1.08), <0.01 | 0.12(-1.38, 1.63), 0.87 | -0.06(-1.12, 1.00), 0.91 |

Notes: cells show β (95% confidence intervals), and p-values; sample sizes in the different periods were (male/female), model a: 334 males and 354 females in all periods; model b: 2 (593/627); 2–4 (561/580); 4–7 (574/615); 7–11 (573/622); 11–15 (543/588); 15–20 (500/555)

*adjusted for weight at 2 years

Pubertal timing was categorised in four groups using prospectively ascertained physical examinations of genitalia development and voice breaking status at 14 years (males) or age at menarche (females) as previously described.^37^ Father’s occupational class (Registrar General’s social classification) was also included as a categorical term, from 1(professional) to 6(unskilled) and was prospectively ascertained at 4 years, or 11(n=5) or 15(n=3) if unavailable then.

Supplementary Table 6. Mean differences in android and gynoid fat mass (kg) per standard deviation increase in: a) birth weight or weight gain velocity (adjusted for weight and height at the beginning of each period and concurrent height gain velocity); b) height at 2 years or height gain velocity (adjusted for weight and height at the beginning of each period and concurrent weight gain velocity)

|  | a)  Birth weight  and weight gain |  |  |  | b)  Height at 2 years  and height gain |  |  |
| --- | --- | --- | --- | --- | --- | --- | --- |
| Outcome  and period  of weight  gain (years) | Males | Females | P# | Outcome  and period  of height  gain (years) | Males | Females | P# |
| Android fat mass (kg) |  |  |  |  |  |  |  |
| Birth weight | 0.00(-0.07, 0.07), 0.93 | -0.01(-0.08, 0.06), 0.77 | 0.89 |  |  |  |  |
| 0-2* | 0.07(-0.01, 0.15), 0.09 | 0.00(-0.09, 0.08), 0.97 | 0.22 | 2* | -0.02(-0.10, 0.07), 0.72 | 0.05(-0.03, 0.14), 0.20 | 0.25 |
| 2–4 | 0.00(-0.09, 0.10), 0.94 | 0.10(0.01, 0.20), 0.03 | 0.14 | 2–4 | -0.12(-0.23, -0.01), 0.03 | -0.05(-0.16, 0.05), 0.33 | 0.40 |
| 4–7 | 0.23(0.14, 0.31), <0.01 | 0.08(-0.01, 0.17), 0.09 | 0.02 | 4–7 | -0.03(-0.13, 0.07), 0.54 | 0.07(-0.03, 0.17), 0.15 | 0.15 |
| 7–11 | 0.18(0.09, 0.28), <0.01 | 0.25(0.15, 0.35), <0.01 | 0.29 | 7–11 | -0.02(-0.10, 0.06), 0.64 | -0.03(-0.12, 0.06), 0.47 | 0.83 |
| 11–15 | 0.19(0.08, 0.30), <0.01 | 0.15(0.07, 0.23), <0.01 | 0.56 | 11–15 | -0.11(-0.20, -0.01), 0.04 | 0.03(-0.06, 0.13), 0.52 | 0.05 |
| 15-20 | 0.21(0.11, 0.30), <0.01 | 0.22(0.13, 0.30), <0.01 | 0.87 | 15-20 | -0.03(-0.15, 0.10), 0.66 | -0.04(-0.13, 0.05), 0.41 | 0.91 |
| Gynoid fat mass (kg) |  |  |  |  |  |  |  |
| Birth weight | 0.10(0.02, 0.17), 0.01 | 0.06(-0.04, 0.16), 0.25 | 0.56 |  |  |  |  |
| 0-2* | 0.12(0.04, 0.20), 0.01 | 0.05(-0.07, 0.17), 0.45 | 0.33 | 2* | 0.08(-0.01, 0.16), 0.07 | 0.07(-0.05, 0.19), 0.24 | 0.93 |
| 2–4 | 0.02(-0.07, 0.12), 0.65 | 0.19(0.06, 0.33), <0.01 | 0.05 | 2–4 | -0.06(-0.18, 0.05), 0.28 | 0.06(-0.10, 0.21), 0.48 | 0.23 |
| 4–7 | 0.27(0.17, 0.36), <0.01 | 0.09(-0.03, 0.22), 0.15 | 0.04 | 4–7 | -0.06(-0.16, 0.05), 0.29 | 0.23(0.09, 0.37), <0.01 | <0.01 |
| 7–11 | 0.17(0.07, 0.27), <0.01 | 0.23(0.09, 0.38), <0.01 | 0.51 | 7–11 | 0.03(-0.06, 0.11), 0.58 | 0.02(-0.11, 0.15), 0.79 | 0.93 |
| 11–15 | 0.18(0.06, 0.29), <0.01 | 0.26(0.15, 0.38), <0.01 | 0.33 | 11–15 | -0.14(-0.25, -0.04), 0.01 | 0.16(0.02, 0.30), 0.02 | <0.01 |
| 15-20 | 0.27(0.17, 0.37), <0.01 | 0.29(0.16, 0.41), <0.01 | 0.83 | 15-20 | 0.12(-0.01, 0.25), 0.07 | -0.05(-0.17, 0.08), 0.47 | 0.10 |

Notes: cells show β (95% confidence intervals), and p-values; sample sizes in the different periods were (male/female) a) birth weight (745/808); 0–2 (592/625); 2–4 (561/580); 4–7 (574/615); 7–11 (573/622); 11–15 (543/588); 15–20 (500/555); b): 2 (593/627); 2–4 (561/580); 4–7 (574/615); 7–11 (573/622); 11–15 (543/588); 15–20 (500/555); model b: 334 males and 354 females in all periods

#p(sex interaction term)

*Model a) adjusted for height at 2 years; b) adjusted for weight at 2 years
